# Updating international consensus on best practice in care of the dying: A Delphi study

**DOI:** 10.1177/02692163231152523

**Published:** 2023-02-03

**Authors:** Tamsin McGlinchey, Rebecca Early, Stephen Mason, Carl Johan-Fürst, Lia van Zuylen, Susie Wilkinson, John Ellershaw

**Affiliations:** 1 Palliative Care Unit, University of Liverpool, Liverpool, UK; 2 The Institute for Palliative Care, Lund University and Region Skåne, Lund, Sweden; 3 Department of Medical Oncology, Amsterdam University Medical Centre’s, Cancer Centrum Amsterdam, Amsterdam, The Netherlands

**Keywords:** Palliative care, terminal care, quality of care, consensus development, Delphi method

## Abstract

**Background:**

Good care of the dying has been defined as being able to die in the place of your choice, free from pain, cared for with dignity and supported by the best possible care. This definition underpinned the development of the ‘10/40 Model’ of care for the dying, in 2013. The model includes 10 ‘Key Principles’ that underpin 40 ‘Core Outcomes’ of care. It was necessary to update consensus on the 10/40 Model to ensure that it remains clinically relevant and applicable for practice.

**Aim:**

Update international consensus on the content of the 10/40 Model.

**Design:**

Delphi study utilising questionnaire completion; each round informed the need for, and content of the next. Free text comments were also sought. Three rounds of Delphi were undertaken.

**Setting/participants:**

A total of 160 participants took part in round 1, representing 31 countries; 103 in round 2 and 57 in round 3. Participants included doctors, nurses, researchers and allied health professionals, with over 80% working predominantly in palliative care (general/specialist not specified).

**Results:**

Minor amendments were made to seven items related to: recognition of the dying phase, ongoing assessment of the patient’s condition, communication with patients about the plan of care and care in the immediate time after the death of a patient. Results supported the addition of a sub core outcome for care provided after death.

**Conclusion:**

The updated 10/40 Model will guide the delivery of high-quality care for dying patients regardless of the location of care. Further work should focus on increasing lay participation and participation from low income and culturally diverse countries.

**What is already known about the topic?**

- The 10/40 Model was developed by The International Collaborative for Best Care for the Dying Person in 2013 and represents internationally agreed best practice in care of the dying, incorporating 10 Key Principles and 40 Core Outcomes for care
- The International Collaborative is committed to a 5-year quality improvement cycle to review international consensus on what constitutes best care for the dying patient.

**What this paper adds?**

- Following a three-round international Delphi, 8/10 Key Principles and 35/40 Core Outcomes achieved consensus at the first round and remain in the model without amendment.
- Amendments were made to 2/10 key principles and 5/40 Core Outcomes, which encompassed aspects of care including: recognition of the dying phase, ongoing assessment of the patient’s condition, communication with patients about the plan of care and care in the immediate time after the death of a patient.

**Implications for practice, theory or policy**

- The 10/40 Model, updated in 2021, will guide the delivery of high-quality care for all dying patients regardless of the location of care.
- The 10/40 Model can be used to develop locally relevant clinical documentation to record patient outcomes based on internationally agreed best practice.
- The 10/40 Model can be a useful tool for healthcare providers to inform the delivery of end-of-life-care at the bedside, and provide a framework for documentation and monitoring.

## Background

Good care at the end of life has been defined as being able to die in the place of your choice, free from pain, cared for with dignity and supported by the best possible care.^1^ Despite knowledge of what a ‘good death’ should look like, many people still receive end of life care that is less than optimal.^1^ Due to increases in life expectancy and prevalence of chronic illness, many more people are expected to die with serious health related suffering,^2^ yet palliative care and the relief of suffering are some of the most neglected areas of global health.^3^ Even in the Western tradition where palliative care is an established medical speciality, the realities of everyday practice can reveal underlying social, moral and organisational tensions that challenge the provision of optimal care at the end of life.^4^ It is imperative that all healthcare providers are enabled to deliver good end of life care wherever patients die, especially where access to specialist palliative care support may be limited, including low and middle-income countries.^5^

Ensuring a world where all people experience a good death as an integral part of their individual life, supported by the very best personalised care is the central vision of the International Collaborative for Best Care for the Dying Person (The Collaborative). The Collaborative was established in 2013 by a group of clinicians and researchers from 12 countries following participation in a European Union Seventh Framework funded study, OPCARE9. OPCARE9 systematically evaluated the evidence base for the care of dying cancer patients across a range of healthcare environments and diverse cultures, across five themes: Signs and symptoms of approaching death, end of life decisions, complementary comfort care, psychological and psychosocial support and voluntary service.^6^ Although OPCARE9 found limited research evidence, findings advanced consensus positions on optimum care for dying cancer patients and developed innovative research protocols to address identified knowledge gaps and needs. Drawing on findings from OPCARE9, as well as wider national and international evidence and consensus opinion, 10 key elements of care for the dying patient were identified.^1^ In 2013 one of the first initiatives of the newly established Collaborative was to incorporate these 10 key elements into a model of documentation and care delivery for best care for the dying, called the 10/40 Model. The 10/40 Model comprises 10 Key Principles of care that underpin 40 Core Outcomes which organisations can use to develop clinical documentation to promote consistent, equitable and individualised care for every patient, regardless of diagnosis or place of care.^7^

The 10/40 model has been adopted and adapted across a range of different care providing organisations across 11 countries linked through the Collaborative. In some of these countries, the content of the 10/40 model has been used to inform national guidance in palliative and end of life care with government health departments recommending the use of developed clinical care plans that have been derived from the 10/40 model. For example, The Norwegian care plan ‘Last Days of Life’ is included as an example of good practice in the Norwegian Action Programme for Palliative Care in Cancer Care,^8^ as well as in the Norwegian Health Directorate specific guidance for Palliative Care in the Final Days of Life.^9^ In Sweden, the Swedish Palliative Care Guide, a national initiative to improve care provided at the end of life, is included in the Swedish National Palliative Care Guidelines.^10^ There are also emerging examples of where the outcomes within the 10/40 model have been used within a research framework, such as a recent study from the Netherlands looking at prophylactic medication prescribing for death rattle. This study included secondary outcomes, which were assessed using data taken directly from care plans derived from the outcomes in the 10/40 Model, on the assessment and management of care and symptoms in the last hours or days of life. The care plans facilitated consistent documentation, demonstrating how outcomes from the 10/40 model can be used in comparative studies to illustrate the impact of interventions on the care of dying patients.^11^

Since 2013 the 10/40 Model has evolved, with modifications and amendments made following clinical consensus and agreement by members of the International Collaborative. Further robust underpinning is required prior to further international dissemination and use of the 10/40 Model, to ensure that it is evidence based and applicable for practice. As a way to establish the 10/40 model as part of a research framework, a Delphi study was undertaken to confirm international consensus on the content of the 10/40 model.

## Aim

To update international consensus on the content of the clinical framework within the 10/40 Model, including the 10 key principles and 40 core outcomes.

This study was guided by the following objectives:

1. Conduct rounds of Delphi questionnaire to assess the relevance and applicability of the key principles and core outcomes in the 10/40 model
2. Make recommendations for amendments following expert review as required
3. Gain international agreement for the final content of the updated 10/40 Model

## Design

This study engaged Delphi methodology which facilitates a consensus building approach to the collection and synthesis of data from a group of knowledgeable experts,^12^ to formulate a consensus when there is limited or equivocal evidence.^13,14^ Delphi studies have been used widely to drive the development of best practice guidelines in palliative care, and have been useful in facilitating international collaborations.^12^ The ‘Guidance on Conducting and REporting DElphi Studies in palliative care’ (CREDES)^15^ was used to ensure robust method and reporting.

The study was undertaken using three rounds of Delphi questionnaire. This study did not pre-determine the number of Delphi rounds a-priori. Each round was informed by the results from the previous, until a consensus was reached on the final content. Distribution and return of questionnaires was anonymous, promoting ‘independent objectivity’.^16^

Each round of Delphi was developed for online completion using Google Forms to facilitate dissemination and promote greater participation. Data were collected between February and June 2021.

### Setting

The focus of the Delphi was on outcomes of care for patients in the last hours or days of life, regardless of their diagnosis or place of care. Participants were sought from across all settings where dying patients are cared for.

### Population

The target population were healthcare professionals who have experience in the care of dying dying patients and their families. This Delphi did not apply strict inclusion/exclusion criteria in order to broaden participation and encourage a wide range of expertise. Following the initial sampling approach (see below), the idea was that participants would ‘self-select’ based on their expertise and/or interest in improving the care of dying patients, and their willingness to participate in the Delphi study.

### Sampling approach

Purposive and Snowball sampling were used to recruit participants.^17^ Potential participants in were initially identified through their affiliation with the International Collaborative, as individuals with a breadth of expertise and knowledge about palliative care and care of the dying (purposive sampling) representing a wide geographical/international spread (22 Countries across Europe, South America, Oceania and Asia). The Collaborative comprises a multi-disciplinary group of clinicians, researchers and other professionals and volunteers working in palliative and end-of-life care. Membership of the Collaborative is open to any persons (or groups) that have an interest in improving the care of dying patients.

It was acknowledged that sampling through the Collaborative may introduce a risk of acceptability bias. Considering this, all initial recipients of the Delphi invitation were encouraged to forward this on to any contact, local or wider, that they felt may be interested in contributing to this study (snowball sampling). Participation in the study was also advertised via the European Association for Palliative Care Blog.^18^

### Recruitment

All potential participants were provided with an information sheet and asked to complete an electronic consent form prior to each Delphi round. To ensure confidentiality and enable individual review, completed questionnaires were given a unique identifier, with the link between the identifier and participant destroyed following the final round of Delphi.

### Data collection

#### Delphi Round 1: Establishing levels of agreement for existing ‘items’ included in the 10/40 Model

The Round 1 Delphi questionnaire included 50 ‘items’ in total; 10 Key Principles and 40 Core Outcomes. Participants were asked to rate their agreement with each ‘item’ using a 5-point Likert scale as follows: 1 – strongly disagree, 2 – disagree, 3 – neither agree nor disagree, 4 – agree and 5 – strongly agree. The Delphi questionnaire provided the opportunity for participants to suggest new items, or submit any other comments about the items currently included in the 10/40 model. Free text comments boxes were included after each item for participants to make specific comments about individual items, but there was also a free text comments box at the end of each section that specifically asked participants to highlight anything that may currently be missing from that particular section. The Delphi Round 1 questionnaire is available in supplementary materials.

#### Round 2 Delphi: Levels of agreement for revised items following Round 1

A Round 2 Delphi questionnaire was distributed to all Round 1 respondents, to assess levels of agreement for amended/additional items (see Table 4). Levels of agreement were determined using the same Likert scale as Delphi Round 1. Free text comments were also sought from participants, as in Round 1, to ask for comments and suggestions on existing items, or offer suggestions for new areas/items for inclusion.

#### Round 3 Delphi: Final comments and suggestions for remaining item

A third round Delphi questionnaire was distributed to all Round 2 respondents to further explore perceptions and comments on items which did not reach the threshold for inclusion when presented in Round 2. This questionnaire allowed free text comments only. Respondents had the opportunity to explain their thoughts and suggest alternative wording. Free text comments allow for a richer and more detailed explanation of participants thoughts than could be obtained through the allocation of a score alone. Comments were then collated for review by the senior research team.

### Data analysis

#### Round 1 Delphi

Percentages, median values and inter quartile ranges (IQRs) were calculated for each ‘item’ on the questionnaire to describe the spread of answers. These values were used to determine the ‘level of agreement’ across participants, for each ‘item’^19^:

- ‘Very high agreement’ – median 5; percentage agreement ⩾80%; IQR 0
- ‘High agreement’ – median 4/5; percentage agreement ⩾80%; IQR 1
- ‘Moderate agreement’ – median ⩽4; percentage agreement 60–79%; IQR 1
- ‘Low agreement’ – median <4; percentage agreement <60%; IQR >1

Due to the likely acceptability bias among the Delphi participants, the threshold for inclusion was set at ‘very high’ agreement for Round 1. Those items that reached a ‘very high’ level of agreement were deemed to have reached a consensus and remained in the Model. For items that did not receive a ‘very high’ level of agreement, free text comments were reviewed by the project team and individual items were revised for inclusion on a second round Delphi questionnaire.

#### Round 2 Delphi

Percentages, median values and inter quartile ranges (IQRs) were calculated for each ‘item’ on the questionnaire to determine levels of agreement as in Round 1.

Following the strict threshold for inclusion imposed in the previous round, in Round 2 this was set to items receiving a ‘high’ or ‘very high’ level of agreement, reflecting previous Delphi studies in palliative care^19,20^ Items receiving ‘very high’ or ‘high’ levels of agreement were deemed to have reached the threshold for inclusion in the final updated model. Items that did not reach this threshold were further explored through Round 3 Delphi.

#### Round 3 Delphi

Free text comments were reviewed by the study researchers (TM/RE) and the comments were then categorised under three pre-determined themes: ‘question wording’, ‘acceptability’, ‘alternative wording suggestion’. Comments categorised under these themes were summarised to provide an overview of the main thoughts and perceptions for review by the senior research team (JE/CJ-F/LvZ/SW). The senior research team made the final decision on the wording of the item for inclusion.

### Ethical review

The study gained ethical approval from the University of Liverpool Research Ethics Committee (reference number: 6401).

## Results

### Delphi rounds

#### Participation

Table 1 shows participation in the three rounds of Delphi by age, gender and profession, including whether the participant worked predominantly in Palliative Care. Table 1 also provides a breakdown of participation per continent, for each round.

**Table 1.** Demographics.

Demographics	Round 1		Round 2		Round 3	
	Frequencies (*n* = 160)		Frequencies (*n* = 103)		Frequencies (*n* = 57)	
Age						
Median	50		50		52	
Interquartile range	42–58 (min–max: 28–85)		50–56 (min–max: 29–75)		44–60 (min–max: 29–75)	
Gender						
Female	76% (122)		71% (73)		68% (39)	
Male	24% (38)		29% (30)		32% (18)	
Profession						
Doctor	48% (76)		54% (56)		53% (30)	
Nurse	32% (51)		28% (29)		23% (13)	
Allied health professional	4% (6)		4% (4)		4% (2)	
Academic (research)	9% (15)		7% (7)		11% (6)	
Educator	1% (2)		2% (2)		4% (2)	
Other (specify)*	6% (10)*		5% (5)**		7% (4)***	
Work predominantly in palliative care						
Yes	82% (131)		85% (88)		88% (50)	
No	18% (29)		15% (15)		12% (7)	
Participation by continent	Round 1		Round 2		Round 3	
	%	(*n* = 160)	%	(*n* = 103)	%	(*n* = 57)
Europe (France, Germany, Greece, Iceland, Ireland, Italy, Netherlands, Norway, Portugal, Slovenia, Spain, Sweden, Switzerland and United Kingdom)	57	91	62	64	65	37
South America (Argentina, Brazil, Chile, Colombia, El Salvador, Peru and Uruguay)	17	27	17	18	14	8
Oceania (Australia and New Zealand)	14	23	22	11	14	8
Asia (India, Japan and Malaysia)	6	10	10	6	7	4
North America (Canada and United States)	4	5	2	2	–	0
Africa (South Africa, South Sudan and Uganda)	3	4	2	2	–	0
Total	100	160	100	103	100	57

Other professions specified as: Psychology***, Social Worker***, Volunteer*, Occupational Therapist***, Retired Nurse and Academic**, Palliative Care Advisor**.

* Round 1; **Round 1 and 2; ***Round 1, 2 and 3.

#### Round 1 Delphi results

Table 2 presents the Round 1 Delphi results. This table includes the level of agreement that was reached for each of the 10 Key Principles and 40 Core Outcomes included in the 10/40 Model. Key Principles/Core Outcomes that reached a ‘Very High’ level of agreement were deemed to have reached consensus.

**Table 2.** Amendments to Round 2 Delphi questionnaire, following free text comments from Round 1.

Section 1: key principles	
Comments received: key principles 1–10	
Comments included concerns that the 10:40 model was aimed more towards inpatient care rather than supporting those at home/community/other non-inpatient care setting. Specifically, respondents highlighted that specifying ‘at least 1 doctor and 1 nurse’ (Key Principle 1) could present a challenge if access to ‘senior doctor’ is limited due to availability or the constitution of the healthcare team within the individual care setting/country. Additionally, comments highlighted that ‘regular 4 hourly re-assessment of patients’ (Principle 9) may not always possible in non-inpatient settings, or in areas of low resource.	
Original wording	Suggested amendment
Key principle 1: recognition that the person is in the last few days and hours of life should be made by the multidisciplinary team (minimum doctor and nurse) and documented by the senior doctor responsible for the person’s care	Key principle 1: recognition that the person is in the last few days and hours of life should be made by the multidisciplinary team (ideally a doctor and a nurse) and documented by a senior healthcare professional responsible for the person’s care
Key principle 9: there should be regular reassessments of the dying person at least every 4 h and review by the multidisciplinary team at least every 48 h	Key principle 9: there should be regular reassessments of the dying person at least every 4 h in in-patient settings, or at each contact in the community setting. Review by the multidisciplinary team at least every 48 h
Section 2: core outcomes	
Comments received: initial assessment section (core outcomes 1.1–1.9)	
Comments received related to issues with communication with the dying person (Core Outcomes 1.1, 1,7), specifically that a person may be too confused/delirious/tired/semi-conscious to engage in the conversation. For example, the focus on the patient being an ‘active part’ in communication might not be possible in many cases. Other comments reflected concerns that some items were aimed more towards inpatient care rather than supporting those at home/community/other non-inpatient care setting (Core Outcomes 1.4, 1.9).	
Original wording	Suggested amendments
1.1: The person is able to take a full and active part in communication	1.1: The person takes a full and active part in communication.
1.4: The relative or carer or advocate has had a full explanation of the facilities available to them. A facilities leaflet has been given.	1.4: The relative or carer or advocate has had a full explanation of the facilities/services available to them. A facilities/services leaflet has been given.
1.7: The person can express an understanding of their individualised current plan of care	1.7: The person expresses an understanding of their individualised current plan of care.
1.9: The medical team that supports the person in their usual place of residence is notified that the person is thought to be dying	1.9: All healthcare teams that support the person are notified that the person is thought to be dying.
Comments received: care after death section (core outcomes 5.1–5.3)	
Respondents highlighted a gap in the current outcomes, specifically commenting that there was not enough emphasis on the need to facilitate cultural, religious or spiritual rituals and practices surrounding the care of the deceased body (Core Outcome 5.1). Comments also suggested the need for an additional Core Outcome within the Care After Death Section, to focus on bereavement/grief support for the patient’s family.	
Original wording	Suggested amendments
5.1. Care of the deceased body is undertaken according to policy and procedure	5.1. Care of the deceased body is undertaken according to religious/spiritual practices and local policy and procedure
Care after death: additional outcome: the relative or carer or advocate is given written information on bereavement and the bereavement services available to them.	

All items reached a ‘Very High’ or ‘High’ level of agreement, illustrating overall agreement with the content of the 10/40 Model. Eight out of 10 Key Principles (80%) and 35/40 Core Outcomes reached a ‘Very High’ level of agreement. Items that reached a ‘Very High’ level of agreement were deemed to have reached consensus, and were taken forward into the updated 10/40 Model unchanged.

From the free text comments received, some wording changes were suggested for those items that received a ‘High’ level of agreement, specifically:

- Key Principles: 1 and 9
- Core Outcomes: 1.1, 1.4, 1.7, 1.9 and 5.1

An additional Core Outcome was suggested for ‘Section 5, Care After Death’ to include signposting for bereavement support (Table 3).

**Table 3.** Round 1 results for the 10 key principles and 40 core outcomes.

Item	Median score (1–5 scale)	IQR	% Agreement (score of 4/5)	Level of agreement
10 key principles				
1. Recognition that the person is in the last few days and hours of life should be made by the multidisciplinary team (minimum doctor and nurse) and documented by the senior doctor responsible for the person’s care.	5	1	87	High
2. Communication of the recognition of dying should be shared with the person where possible and deemed appropriate and with those important to them	5	0	97	Very high
3. The dying person and those important to them - relative or carer or advocate should have the opportunity to discuss their wishes, concerns, feelings, faith, beliefs, values	5	0	100	Very high
4. Anticipatory prescribing for symptoms that can be expected (e.g. pain) should be available	5	0	99	Very high
5. All clinical interventions are reviewed in the best interest of the individual person	5	0	98	Very high
6. There should be a review of hydration needs including the commencement, continuation or cessation of clinically assisted (artificial) hydration	5	0	93	Very high
7. There should be a review of nutritional needs including the continuation or cessation of clinically assisted (artificial) nutrition	5	0	94	Very high
8. There should be a full discussion of the plan of care with the dying person where possible and deemed appropriate and with those important to them/relative or carer or advocate	5	0	98	Very high
9. There should be regular reassessments of the dying person at least every 4 h and review by the multidisciplinary team at least every 48 h	5	1	82	High
10. Care for the dying person and those important to them/relative or carer or advocate immediately after death is dignified and respectful	5	0	98	Very high
40 core outcomes				
1.1. The person is able to take a full and active part in communication	5	1	82	High
1.2. The relative or carer or advocate is able to take a full and active part in communication	5	0	94	Very high
1.3. The clinical team have up to date contact information for the relative or carer or advocate	5	0	99	Very high
1.4. The relative or carer or advocate has had a full explanation of the facilities available to them. A facilities leaflet has been given.	5	1	90	High
1.5. The person is given the opportunity to discuss what is important to them at this time for example, their wishes, concerns, feelings, faith, beliefs and values	5	0	98	Very high
1.6. The relative or carer is given the opportunity to discuss what is important to them at this time for example, their wishes, concerns, feelings, faith, culture, beliefs and values	5	0	98	Very high
1.7. The person can express an understanding of their individualised current plan of care	5	1	89	High
1.8. The relative or carer can express an understanding of the individualised current plan of care	5	0	97	Very high
1.9. The medical team that supports the person in their usual place of residence is notified that the person is thought to be dying	5	0	90	Very high
2.1. The person has medication prescribed on an ‘as required’ basis for all of the following five symptoms which may develop in the last few days and hours days of life: pain, nausea and/or vomiting, dyspnoea, restlessness and agitation and respiratory tract secretions	5	0	98	Very high
2.2. Equipment is available for the person to support a continuous subcutaneous (or intravenous) infusion of medication where required	5	0	91	Very high
2.3. All current interventions have been reviewed for example, routine blood tests, medications and recording of routine vital signs and oxygen therapy	5	0	93	Very high
2.4. The person’s resuscitation status has been reviewed	5	0	93	Very high
2.5. Implantable Cardioverter Defibrillator (ICD) status is reviewed	5	0	88	Very high
2.6. The need for clinically assisted (artificial) hydration is reviewed	5	0	93	Very high
2.7. The need for clinically assisted (artificial) nutrition is reviewed	5	0	91	Very high
3.1. The Person’s mouth is moist and clean	5	0	98	Very high
3.2. The Person’s skin integrity is assessed	5	0	100	Very high
3.3. The Person’s hygiene needs are assessed	5	0	100	Very high
4.1. The person does not have pain	5	0	97	Very high
4.2. The person is not agitated	5	0	98	Very high
4.3. The person does not have respiratory tract secretions	5	1	94	High
4.4. The person does not have nausea	5	0	96	Very high
4.5. The person is not breathless	5	0	98	Very high
4.6. The person is not vomiting	5	0	98	Very high
4.7. The person does not have urinary problems	5	0	97	Very high
4.8. The person does not have bowel problems	5	0	90	Very high
4.9. The person does not have other symptoms	5	0	94	Very high
4.10. The person’s comfort and safety regarding the administration of medication is maintained	5	0	99	Very high
4.11 The person receives fluids to support their individual needs	5	1	79	High
4.12. The person’s mouth is moist and clean	5	0	99	Very high
4.13. The person’s skin integrity is maintained	5	0	96	Very high
4.14. The person’s personal hygiene needs are met	5	0	99	Very high
4.15. The person receives their care in a physical environment adjusted to support their individual needs	5	0	99	Very high
4.16. The person’s psychological and spiritual well-being is supported	5	0	99	Very high
4.17. The well-being of the relative or carer or advocate attending the person is supported	5	0	99	Very high
5.1. Care of the deceased body is undertaken according to policy and procedure	5	0	94	Very high
5.2. The relative or carer or advocate can express an understanding of what they will need to do next and are given the relevant written information	5	0	96	Very high
5.3. The multi professional team that supported the person in their usual place of residence is notified of the person’s death	5	0	96	Very high
5.4. The person’s death is communicated to appropriate services across the organisation	5	0	94	Very high

#### Round 2 Delphi results

A Round 2 Delphi questionnaire was sent out to all Round 1 participants, to assess levels of agreement for the amended/additional items. Table 4 illustrates the level of agreement received for each of the seven revised items and the one additional item.

**Table 4.** Levels of agreement for the amended/additional items in the Round 2 Delphi questionnaire.

Original wording	Suggested amendment	Median score (1–5 scale)	IQR	% Agreement (score of 4/5)	Level of agreement
Section 1: key principles					
Key Principle 1: Recognition that the person is in the last few days and hours of life should be made by the multidisciplinary team (minimum doctor and nurse) and documented by the senior doctor responsible for the person’s care	Key Principle 1: Recognition that the person is in the last few days and hours of life should be made by the multidisciplinary team (ideally a doctor and a nurse) and documented by a senior healthcare professional responsible for the person’s care	5	1	86%	High
Key Principle 9: There should be regular reassessments of the dying person at least every 4 h and review by the multidisciplinary team at least every 48 h	Key Principle 9: There should be regular reassessments of the dying person at least every 4 h in in-patient settings, or at each contact in the community setting. Review by the multidisciplinary team at least every 48 h	5	1	91%	High
Section 2: core outcomes					
1.1: The person is able to take a full and active part in communication	1.1: The person takes a full and active part in communication.	4	2	75%	Low
1.4: The relative or carer or advocate has had a full explanation of the facilities available to them. A facilities leaflet has been given.	1.4: The relative or carer or advocate has had a full explanation of the facilities/services available to them. A facilities/services leaflet has been given.	5	1	93%	High
1.7: The person can express an understanding of their individualised current plan of care	1.7: The person expresses an understanding of their individualised current plan of care.	4	1	84%	High
1.9: The medical team that supports the person in their usual place of residence is notified that the person is thought to be dying	1.9: All healthcare teams that support the person are notified that the person is thought to be dying.	5	1	96%	High
5.1. Care of the deceased body is undertaken according to policy and procedure	5.1. Care of the deceased body is undertaken according to religious/spiritual practices and local policy and procedure	5	0	97%	Very High
Care after death: additional core outcome: The relative or carer or advocate is given written information on bereavement and the bereavement services available to them.		5	1	93%	High

Round 2 results confirmed agreement with amended wording for the following items: Key Principles: 1, 9; Core Outcomes: 1.4, 1.7, 1.9, 2.1. High agreement was also reached for the additional Core Outcome in section 5, Care After Death. These items were deemed to have sufficient agreement across the participant group to be taken forward into the updated Model.

For Core Outcome 1.1 (*The person is able to take a full and active part in communication*), these results suggested that further interrogation was required, as the suggested wording change reached a low level of agreement.

#### Round 3 Delphi results

The Round 3 Delphi focussed on Core Outcome 1.1 to gain deeper insight into the reasons for a low agreement with the amended wording. Participants were asked to provide comments on the current and revised wording of Core Outcome 1.1 and were also given the opportunity to make suggestions for alternative wording:

- Current wording: The person is able to take a full and active part in communication
- Revised wording: The person takes a full and active part in communication

##### Summary of comments received

**Figure 1.**
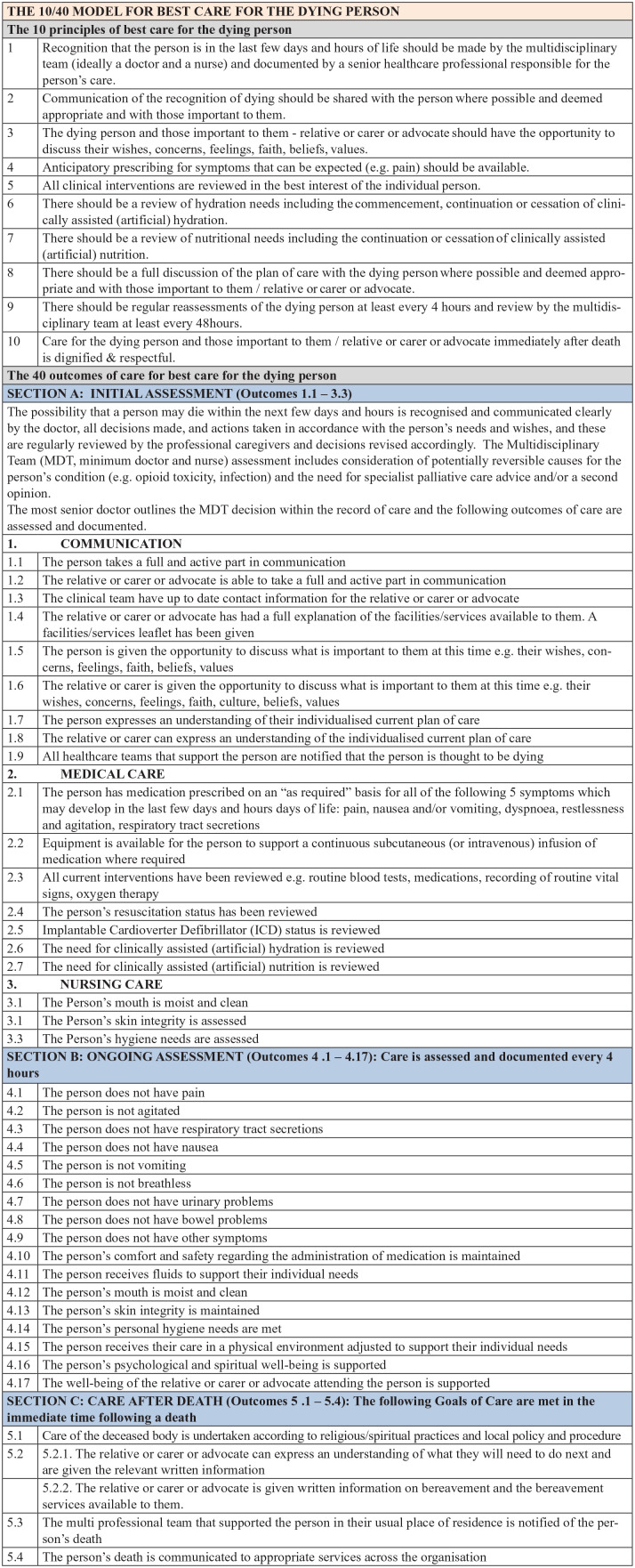
Updated version of the 10/40 model following three rounds of Delphi.

The majority of comments suggested that the revised wording was preferred over the original wording. However, comments also highlighted specific concerns that this outcome may not be achievable for many patients due to reasons such as, a deteriorating clinical condition, challenges or barriers to engaging in communication or the patient’s decision not to participate in communication. Reflecting these concerns, comments also highlighted the importance of having a clear process of documentation so that information about why this outcome may not have been achieved can be recorded at the point of care delivery. The decision by the research team was to include the *revised* wording of Core Outcome 1.1 in the updated version of the 10/40 Model. Figure 1 presents the updated version of the 10/40 Model following the three rounds of Delphi.

## Discussion

### Main findings from the study

This Delphi updated the 10/40 Model for the best care for dying patients though international consensus. Following Delphi Round 1 participants reached consensus on 8/10 Key Principles and 35/40 Core Outcomes, meaning these items remained in the 10/40 Model without amendment. For the remaining two Key Principles and five Core Outcomes, another two rounds of Delphi further explored participants’ thoughts, including seeking agreement for suggested amendments. Minor amendments were made to the model overall, suggesting that the initial work by the International Collaborative into what constitutes good end-of-life-care for dying patients is still relevant and applicable to practice. The 10/40 Model will be reviewed and updated (if required), as part of a 5-year continuous quality improvement cycle by the International Collaborative.

### Strengths and limitations

This Delphi study was conducted as part of the research activities of the International Collaborative for Best Care for the Dying Patient, and benefitted from the expertise of its world-wide, multi-professional membership, not only in project design and management of the study via its co-lead investigators, but through access to the international membership group for initial questionnaire dissemination. A limitation of this study is the lack of responses from multi-disciplinary health care professionals. The majority of participants were doctors predominantly working in palliative care. A methodological limitation of this study was the initial recruitment through the membership of the International Collaborative. Future work to assess the appropriateness and acceptability of the 10/40 Model should seek to maximise participation from other professional groups as well as groups not linked through the International Collaborative, and increase lay participation from patient and public groups. Another limitation is that this study constitutes a majority opinion from a mainly white, European population. Increasing levels of participation from lower income and culturally diverse countries should be a specific focus for future work on the 10/40 Model. For example, existing links within the International Collaborative membership could be more meaningfully explored to open up wider collaboration from these countries into the future, and connect with other national and international organisations. A further limitation is that no formal review of the literature was undertaken to inform the Delphi process.

### What this study adds?

Using Delphi methodology over three rounds, this study confirmed consensus on what constitutes best care for the dying person, resulting in an updated version of the 10/40 model. The Model will be subject to a 5-year continuous quality improvement cycle. Following round 1 of the Delphi only 2/10 Key Principles and 35/40 Core Outcomes did not achieve consensus (or ‘very high’ agreement) to remain in the Model without amendment. Items that did not reach consensus still achieved a ‘high’ level of agreement, which in other Delphi studies has been a threshold for inclusion.^19,20^ However, as the 10/40 Model had already been developed and is viewed as an accepted model of care amongst many members of the International Collaborative, it was decided that unless an item achieved the highest level of agreement in Round 1 Delphi, it would be subjected to further scrutiny by reviewing the free text comments obtained through the Delphi questionnaire. The free text comments received identified important considerations for wording of items, including the way that the 10/40 Model is interpreted and incorporated into end-of-life-care delivery within individual organisations.

Round 1 free-text comments identified concerns that Key Principles 1 and 9 were more aligned to care provided in an inpatient setting, rather than at home, in the community or areas of remote/limited access to a multi-professional healthcare team. For example, the requirement that a patient be reassessed ‘at least every 4 hours’ (Key Principle 9) was highlighted specifically, with comments suggesting a revision of the wording to reflect situations outside the inpatient setting; ‘at least every 4 hours in in-patient settings, or at each contact in the community setting’. For Key Principle 1, specifying that the recognition of dying must be made by ‘at least 1 doctor and 1 nurse’ also posed a challenge for some respondents if access to a ‘senior doctor’ is limited, therefore, an amendment was made to state ‘*ideally* a doctor and a nurse’. It is important that the Key Principles on which the 10/40 Model are based represent a global imperative to promote quality and sustainable palliative care systems,^5^ but that they also reflect the challenge of providing palliative care at all levels of care.^21^

One of the Core Outcomes that decreased in level of agreement following amendment to the wording in Round 2 of the Delphi was Core Outcome 1.1, that the dying person is able to take a ‘full and active part’ in communication. Round 2 comments suggested that a patient in the dying phase may be too confused/delirious/tired/semi-conscious to actively engage in communication, therefore the outcome would not be achieved for a large proportion of individuals. Suggestions for amendment focussed on wording amendments to provide caveats by adding phrases such as ‘if possible’ or ‘if/where appropriate’, allowing for an ‘opt out’ of the outcome where communication proved challenging, rather than acknowledging that it could not be achieved. However, we know clinicians may avoid communication with patients about the end-of-life prior to the patient entering the last days or hours or life. Barriers such as prognostic uncertainty, fear of causing distress, navigating patient readiness and feeling inadequately trained are common themes.^22^ The decision from the senior research team to include outcome 1.1 without a caveat of ‘if possible/where appropriate’ reflected a strong belief that the focus of the outcome should not be distracted from. This was to ensure that the outcome was not diluted, and to avoid distracting from the main focus of the outcome, which was to ensure that communication with the patient was facilitated in the most appropriate way despite their deteriorating condition, and not avoided due to opt out’s that are made possible by including phrases such as ‘if possible/where appropriate’.

Additional comments related to similar concerns that some Core Outcomes would not be achieved for all patients, all the time. However, there is an expectation that not all outcomes will be achieved for 100% of patients despite the best attempts of the healthcare team, and it is important for this to be documented. For example, with regards to symptom control, it has been shown that pain, breathlessness and fatigue are common for many patients at the end-of-life.^23^ It is important that local documentation processes enable important information such as this to be recorded. The information learned from outcomes that are not achieved have the potential to be locally, or nationally transformative in the way that end-of-life-care is provided.

The 40 Core Outcomes within the 10/40 Model reflect important elements of care in the last hours or days of life, which should be included in any local clinical documentation/care plans, tailored to local organisational and cultural contexts and embedded within local governance arrangements. The 10/40 Model can offer a framework to inform how care is delivered to patients, but also serve as a mechanism to monitor care that is delivered. In addition, it can promote quality improvement initiatives and provide outcomes for research in care for the dying. It is important to highlight that ongoing research by Zambrano et al.^24^ (which includes some of the authors of this paper) to develop a core outcome set (COS) for care of the dying, has the potential to complement and inform the ongoing development and use of the 10/40 model. This work aims to achieve consensus opinion on what are the most important outcomes that should be measured in research studies, to assess the quality of care that has been provided to patients and their families at the end of life, for example, after implementation of a model of care such as the 10/40 model. The development of a COS that is specific to care provided in the last hours or days of life will therefore be important for future research in end of life care, such as implementation research into models of care such as the 10/40 model to improve care, and will promote comparative research nationally and internationally.

## Conclusion

This study concludes a three round Delphi study to update international consensus for the content of the 10/40 Model. This study also initiates a 5-year quality improvement programme to continually review and reassess what constitutes best practice in the care of dying patients. As well as gaining consensus on the essential elements of good end-of-life-care, this Delphi also enabled important changes to be made to the Model through collection of free text responses from participants. Future research as part of the 5-year continuous quality improvement programme will also utilise systematic review and scoping review methodology (where appropriate and possible) to ensure that the concepts and ‘items’ within the 10/40 Model continue to represent current evidence in palliative and end of life care.

## Supplemental Material

sj-pdf-1-pmj-10.1177_02692163231152523 – Supplemental material for Updating international consensus on best practice in care of the dying: A Delphi study Click here for additional data file. Supplemental material, sj-pdf-1-pmj-10.1177_02692163231152523 for Updating international consensus on best practice in care of the dying: A Delphi study by Tamsin McGlinchey, Rebecca Early, Stephen Mason, Carl Johan-Fürst, Lia van Zuylen, Susie Wilkinson and John Ellershaw in Palliative Medicine
