# Supplementary material for: Updating international consensus on best practice in care of the dying: A Delphi study
Source: Palliat Med. 2023 Feb 3;37(3):329–42. doi: 10.1177/02692163231152523 (PMC10021119; doi:10.1177/02692163231152523)
Supplement: sj-pdf-1-pmj-10.1177_02692163231152523 – Supplemental material for Updating international consensus on best practice in care of the dying: A Delphi study [file sj-pdf-1-pmj-10.1177_02692163231152523.pdf]

Consensus on the content of the 10/40 model for best care of the dying. Round 1 Delphi Questionnaire

The 10 principles of best care for the dying person

International evidence identifies ten principles of care necessary for achieving best care for the dying person (BMJ 2013). These principles underpin the International Core Elements for Best Care for the Dying Person. These principles are irrespective of the place of care and transcend international and cultural boundaries

Level of agreement with the 10 principles of best care for the dying person

Statements 1 - 10 below represent the current core principles contained in the 10/40 Model developed by the International Collaborative for Best Care for the Dying Person. The model is designed to support dying patients at the end of life. These Principles should underpin the delivery of good end of life care at the bedside.

<u>Instructions for completion:</u>

1. Level of agreement: Against each principle, indicate your level of agreement on a scale of 1 - 5, as to whether this principle underpins good care for dying patients and their families, at the end of life.

2. Free text comments: There is a free text box against each of the 10 principles. Please write any comments you have about that principle in this box. At the end of this section there is a final free text box for you to suggest anything that you feel is currently missing.

1. Recognition that the person is in the last few days and hours of life should be made by the multidisciplinary team (minimum doctor and nurse) and documented by the senior doctor responsible for the person's care

	1	2	3	4	5	
Low Agreement	○	○	○	○	○	High Agreement

Please enter any comments for Principle 1:

____
____
____

2. Communication of the recognition of dying should be shared with the person where possible and deemed appropriate and with those important to them

	1	2	3	4	5	
Low Agreement	○	○	○	○	○	High Agreement

Please enter any comments for Principle 2:

____
____
____

3. The dying person and those important to them - relative or carer or advocate should have the opportunity to discuss their wishes, concerns, feelings, faith, beliefs, values

	1	2	3	4	5	
Low Agreement	○	○	○	○	○	High Agreement

Please enter any comments for Principle 3:

____

____

____

4. Anticipatory prescribing for symptoms that can be expected (e.g. pain) should be available

	1	2	3	4	5	
Low Agreement	○	○	○	○	○	High Agreement

Please enter any comments for Principle 4:

____

____

____

5. All clinical interventions are reviewed in the best interest of the individual person

	1	2	3	4	5	
Low Agreement	○	○	○	○	○	High Agreement

Please enter any comments for Principle 5:

____

____

____

6. There should be a review of hydration needs including the commencement, continuation or cessation of clinically assisted (artificial) hydration

	1	2	3	4	5	
Low Agreement	○	○	○	○	○	High Agreement

Please enter any comments for Principle 6:

____

____

____

7. There should be a review of nutritional needs including the continuation or cessation of clinically assisted (artificial) nutrition

	1	2	3	4	5	
Low Agreement	○	○	○	○	○	High Agreement

Please enter any comments for Principle 7:

____
____
____

8. There should be a full discussion of the plan of care with the dying person where possible and deemed appropriate and with those important to them / relative or carer or advocate

	1	2	3	4	5	
Low Agreement	○	○	○	○	○	High Agreement

Please enter any comments for Principle 8:

____
____
____

9. There should be regular reassessments of the dying person at least every 4 hours and review by the multidisciplinary team at least every 48hours

	1	2	3	4	5	
Low Agreement	○	○	○	○	○	High Agreement

Please enter any comments for Principle 9:

____
____
____

10. Care for the dying person and those important to them / relative or carer or advocate immediately after death is dignified & respectful

	1	2	3	4	5	
Low Agreement	○	○	○	○	○	High Agreement

Please enter any comments for Principle 10:

____
____
____

The 40 outcomes of care for best care for the dying person

The 10 Principles outlined previously underpin the 40 Outcomes of Care for Best Care for the Dying Person, which should be reflected in the outcome measures for any clinical document used at the bedside to support care.

Level of agreement with the 40 outcomes of care for best care for the dying person
The 10:40 Model includes 40 outcomes of care. These outcomes are underpinned by the 10 Principles to guide the documentation of good end of life care at the bedside.

This section of the questionnaire is in three sections:
Section A: Initial Assessment (Outcomes 1.1 - 3.3)
Section B: Ongoing Assessment (Outcomes 4.1 - 4.17)
Section C: Care After Death (Outcomes 5.1 - 5.4)

<u>Instructions for completion:</u>
1. Level of agreement: Against each outcome, indicate your level of agreement on a scale of 1 - 5, as to whether this outcome represents an element of good care for dying patients and their families, at the end of life.

2. Free text comments: There is a free text box against each of the 40 outcomes. Please write any comments you have about this outcome in this box. At the end of each section, there is a final free text box for you to suggest any 'items' that you feel are currently missing from that section.

SECTION A: INITIAL ASSESSMENT (goals of care 1 – 3)

1. COMMUNICATION

Outcome 1.1 The person is able to take a full and active part in communication

	1	2	3	4	5	
Low Agreement	○	○	○	○	○	High Agreement

Please enter any comments for Outcome 1.1:
____
____
____

Outcome 1.2 The relative or carer or advocate is able to take a full and active part in communication

	1	2	3	4	5	
Low Agreement	○	○	○	○	○	High Agreement

Please enter any comments for Outcome 1.2:
____
____
____

Outcome 1.3 The clinical team have up to date contact information for the relative or carer or advocate

	1	2	3	4	5	
Low Agreement	○	○	○	○	○	High Agreement

Please enter any comments for Outcome 1.3:

____
____
____

Outcome 1.4 The relative or carer or advocate has had a full explanation of the facilities available to them. A facilities leaflet has been given

	1	2	3	4	5	
Low Agreement	○	○	○	○	○	High Agreement

Please enter any comments for Outcome 1.4:

____
____
____

Outcome 1.5 The person is given the opportunity to discuss what is important to them at this time e.g. their wishes, concerns, feelings, faith, beliefs, values

	1	2	3	4	5	
Low Agreement	○	○	○	○	○	High Agreement

Please enter any comments for Outcome 1.5:

____
____
____

Outcome 1.6 The relative or carer is given the opportunity to discuss what is important to them at this time e.g. their wishes, concerns, feelings, faith, culture, beliefs, values

	1	2	3	4	5	
Low Agreement	○	○	○	○	○	High Agreement

Please enter any comments for Outcome 1.6:

____
____
____

Outcome 1.7 The person can express an understanding of their individualised current plan of care

	1	2	3	4	5	
Low Agreement	○	○	○	○	○	High Agreement

Please enter any comments for Outcome 1.7:

____
____
____

Outcome 1.8 The relative or carer can express an understanding of the individualised current plan of care

	1	2	3	4	5	
Low Agreement	○	○	○	○	○	High Agreement

Please enter any comments for Outcome 1.8:

____
____
____

Outcome 1.9 The medical team that supports the person in their usual place of residence is notified that the person is thought to be dying

	1	2	3	4	5	
Low Agreement	○	○	○	○	○	High Agreement

Please enter any comments for Outcome 1.9:

____
____
____

2. MEDICAL CARE

Outcome 2.1 The person has medication prescribed on an “as required” basis for all of the following 5 symptoms which may develop in the last few days and hours days of life: pain, nausea and/or vomiting, dyspnoea, restlessness and agitation, respiratory tract secretions

	1	2	3	4	5	
Low Agreement	○	○	○	○	○	High Agreement

Please enter any comments for Outcome 2.1:

____
____
____

Outcome 2.2 Equipment is available for the person to support a continuous subcutaneous (or intravenous) infusion of medication where required

	1	2	3	4	5	
Low Agreement	○	○	○	○	○	High Agreement

Please enter any comments for Outcome 2.2:

____
____
____

Outcome 2.3 All current interventions have been reviewed e.g. routine blood tests, medications, recording of routine vital signs, oxygen therapy

	1	2	3	4	5	
Low Agreement	○	○	○	○	○	High Agreement

Please enter any comments for Outcome 2.3:

____
____
____

Outcome 2.4 The person's resuscitation status has been reviewed

	1	2	3	4	5	
Low Agreement	○	○	○	○	○	High Agreement

Please enter any comments for Outcome 2.4:
____
____
____

Outcome 2.5 Implantable Cardioverter Defibrillator (ICD) status is reviewed

	1	2	3	4	5	
Low Agreement	○	○	○	○	○	High Agreement

Please enter any comments for Outcome 2.5:
____
____
____

Outcome 2.6 The need for clinically assisted (artificial) hydration is reviewed

	1	2	3	4	5	
Low Agreement	○	○	○	○	○	High Agreement

Please enter any comments for Outcome 2.6:
____
____
____

Outcome 2.7 The need for clinically assisted (artificial) nutrition is reviewed

	1	2	3	4	5	
Low Agreement	○	○	○	○	○	High Agreement

Please enter any comments for Outcome 2.7:
____
____
____

3. NURSING CARE

Outcome 3.1 The Person's mouth is moist and clean

	1	2	3	4	5	
Low Agreement	○	○	○	○	○	High Agreement

Please enter any comments for Outcome 3.1:
____
____
____

Outcome 3.2 The Person's skin integrity is assessed

	1	2	3	4	5	
Low Agreement	○	○	○	○	○	High Agreement

Please enter any comments for Outcome 3.2:
____
____
____

Outcome 3.3 The Person's hygiene needs are assessed

	1	2	3	4	5	
Low Agreement	○	○	○	○	○	High Agreement

Please enter any comments for Outcome 3.3:
____
____
____

SECTION B: ONGOING ASSESSMENT (Outcomes 4 .1 – 4.17)

Care is assessed and documented every 4 hours according to the following outcomes below

Outcome 4.1 The person does not have pain

	1	2	3	4	5	
Low Agreement	◯	◯	◯	◯	◯	High Agreement

Please enter any comments for Outcome 4.1:

____

____

____

Outcome 4.2 The person is not agitated

	1	2	3	4	5	
Low Agreement	◯	◯	◯	◯	◯	High Agreement

Please enter any comments for Outcome 4.2:

____

____

____

Outcome 4.3 The person does not have respiratory tract secretions

	1	2	3	4	5	
Low Agreement	◯	◯	◯	◯	◯	High Agreement

Please enter any comments for Outcome 4.3:

____

____

____

Outcome 4.4 The person does not have nausea

	1	2	3	4	5	
Low Agreement	◯	◯	◯	◯	◯	High Agreement

Please enter any comments for Outcome 4.4:

____

____

____

Outcome 4.5 The person is not vomiting

	1	2	3	4	5	
Low Agreement	○	○	○	○	○	High Agreement

Please enter any comments for Outcome 4.5:
____
____
____

Outcome 4.6 The person is not breathless

	1	2	3	4	5	
Low Agreement	○	○	○	○	○	High Agreement

Please enter any comments for Outcome 4.6:
____
____
____

Outcome 4.7 The person does not have urinary problems

	1	2	3	4	5	
Low Agreement	○	○	○	○	○	High Agreement

Please enter any comments for Outcome 4.7:
____
____
____

Outcome 4.8 The person does not have bowel problems

	1	2	3	4	5	
Low Agreement	○	○	○	○	○	High Agreement

Please enter any comments for Outcome 4.8:
____
____
____

Outcome 4.9 The person does not have other symptoms

	1	2	3	4	5	
Low Agreement	◯	◯	◯	◯	◯	High Agreement

Please enter any comments for Outcome 4.9:

____
____
____

Outcome 4.10 The person's comfort and safety regarding the administration of medication is maintained

	1	2	3	4	5	
Low Agreement	◯	◯	◯	◯	◯	High Agreement

Please enter any comments for Outcome 4.10:

____
____
____

Outcome 4.11 The person receives fluids to support their individual needs

	1	2	3	4	5	
Low Agreement	◯	◯	◯	◯	◯	High Agreement

Please enter any comments for Outcome 4.11:

____
____
____

Outcome 4.12 The person's mouth is moist and clean

	1	2	3	4	5	
Low Agreement	◯	◯	◯	◯	◯	High Agreement

Please enter any comments for Outcome 4.12:

____
____
____

Outcome 4.13 The person's skin integrity is maintained

	1	2	3	4	5	
Low Agreement	○	○	○	○	○	High Agreement

Please enter any comments for Outcome 4.13:

____
____
____

Outcome 4.14 The person's personal hygiene needs are met

	1	2	3	4	5	
Low Agreement	○	○	○	○	○	High Agreement

Please enter any comments for Outcome 4.14:

____
____
____

Outcome 4.15 The person receives their care in a physical environment adjusted to support their individual needs

	1	2	3	4	5	
Low Agreement	○	○	○	○	○	High Agreement

Please enter any comments for Outcome 4.15:

____
____
____

Outcome 4.16 The person's psychological and spiritual well-being is supported

	1	2	3	4	5	
Low Agreement	○	○	○	○	○	High Agreement

Please enter any comments for Outcome 4.16:

____
____
____

Outcome 4.17 The well-being of the relative or carer or advocate attending the person is supported

	1	2	3	4	5	
Low Agreement	○	○	○	○	○	High Agreement

Please enter any comments for Outcome 4.17:

____

SECTION C: CARE AFTER DEATH (Outcomes 5 .1 – 5.4)

The following outcomes are met in the immediate time following a death

Outcome 5.1 Care of the deceased body is undertaken according to policy and procedure

	1	2	3	4	5	
Low Agreement	○	○	○	○	○	High Agreement

Please enter any comments for Outcome 5.1:

Outcome 5.2 The relative or carer or advocate can express an understanding of what they will need to do next and are given the relevant written information

	1	2	3	4	5	
Low Agreement	○	○	○	○	○	High Agreement

Please enter any comments for Outcome 5.2:

Outcome 5.3 The multi professional team that supported the person in their usual place of residence is notified of the person's death

	1	2	3	4	5	
Low Agreement	○	○	○	○	○	High Agreement

Please enter any comments for Outcome 5.3:
____
____
____

Outcome 5.4 The person's death is communicated to appropriate services across the organisation

	1	2	3	4	5	
Low Agreement	○	○	○	○	○	High Agreement

Please enter any comments for Outcome 5.4:
____
____
____
